# On-line virtual patient learning: a pilot study of a new modality in antimicrobial stewardship education for pediatric residents

**DOI:** 10.1186/s13104-020-05170-7

**Published:** 2020-07-14

**Authors:** Amer Alshengeti, Kathryn Slayter, Emily Black, Karina Top

**Affiliations:** 1 Present Address: Pediatric Department, College of Medicine, Taibah University, Alqiblatain District, Al-Madinah, 41491 Saudi Arabia; 2 Faculty of Medicine, Department of Medicine, Division of Infectious Diseases, Halifax, NS Canada; 3 Faculty of Graduate Studies, Dalhousie University, Halifax, NS Canada; 4 Canadian Center for Vaccinology, Department of Pharmacy, IWK Health Centre, Halifax, NS Canada; 5 College of Pharmacy, Dalhousie University, Halifax, NS Canada; 6 Department of Pharmacy, IWK Health Centre, Halifax, NS Canada; 7 Department of Pediatrics, Dalhousie University, Halifax, NS Canada; 8 Canadian Center for Vaccinology, IWK Health Centre, Halifax, NS Canada

**Keywords:** Virtual patient, Antimicrobial stewardship, Antimicrobial resistance, Antibiotics education

## Abstract

**Objectives:**

Our objective was to develop and validate a virtual patient (VP) learning module to educate pediatric residents about antimicrobial stewardship (AMS) principles. A VP module on complicated pneumonia was developed by experts in AMS and pediatric infectious diseases using the online platform DecisionSim^™^. Decision points were based on AMS principles (diagnosis, antimicrobial selection, dosing, de-escalation, route, duration). Pediatric residents in all training levels at a tertiary pediatric hospital were recruited to test the VP module. Knowledge was assessed via a multiple choice questionnaire. Mean knowledge scores were compared before, after, and 4 months after completing the module using Generalized Linear Mixed Repeated Measures (RM) Analysis. Resident satisfaction was assessed using a validated questionnaire.

**Results:**

Seven of 24 pediatric residents (Years 1–4) completed the VP module and pre- and post-module questionnaires. Mean knowledge scores before, immediately after and 4 months after the module were 58.2%, 66.6%, and 71.6%, respectively. The change in knowledge across time was significant (p < 0.001). Residents were satisfied with the module as an AMS learning strategy.

## Introduction

Antimicrobial Stewardship Programs (ASP) aim to measure and improve the appropriate use of antimicrobials in order to optimize clinical outcomes and minimize the emergence of antimicrobial resistance (AMR) [1]. Prescriber education is a recommended antimicrobial stewardship (AMS) strategy to increase knowledge about antimicrobial prescribing and AMR, and to stimulate behavioral change in antimicrobial prescribing [1]. Teaching strategies in AMS education include passive and active approaches [1]. The Infectious Diseases Society of America (IDSA) and Society for Healthcare Epidemiology of America (SHEA) recommend incorporating active educational strategies, such as prescriber audit feedback, that are more likely than passive approaches to result in practice changes. [1, 2].

Online learning is one of the suggested methods to actively educate healthcare workers about appropriate antibiotic prescribing, especially in resource limited settings [3, 4]. One online instruction design is Virtual Patient (VP) learning which is defined as “a program that simulates real life clinical scenarios; learners emulate the roles of health care providers to obtain a history, conduct a physical exam, and make diagnostic and therapeutic decisions” [5]. Computerized VP design can be classified into three categories, based the progression of the case: (1) free navigation where the patient’s status remains essentially unchanged as the learner gathers information; (2) linear where the patient’s status evolves over time, but follows the same course regardless of the learner’s decisions; and (3) branching where the patient’s status evolves based on the learner’s decisions [6].VP modules have been used mainly in undergraduate medical education to enhance a variety of outcomes including clinical reasoning and knowledge for various medical conditions [6].

Appropriate antimicrobial prescribing requires good clinical reasoning skills when the clinician collects data, processes the information, develops a differential diagnosis, develops and implements an initial management plan (e.g. decide to prescribe antibiotics, select appropriate antibiotics), and develops a final management plan (e.g. discontinue/modify antibiotics based on patient status and culture results) [7].

Postgraduate trainees play a critical role in shaping antimicrobial utilization patterns in hospitals as part of the front-line clinical team that initiates antimicrobials. However, several studies have reported deviation of prescribing from guidelines for common pediatric infections among pediatric residents [8, 9]. In addition, several studies have shown that the most pediatric residents do not have enough education about AMS during their training [8, 10].

The objectives were to (1) design and validate a VP module to teach pediatric residents the principles of AMS, (2) evaluate the effect of a VP module on pediatric residents’ knowledge about antimicrobial prescribing and AMR and (3) assess residents’ satisfaction with the VP module as an AMS educational strategy.

## Main text

### Methods

This study included two phases: phase 1 involved the development and validation of the VP module, and phase 2 involved: (a) evaluation of the effectiveness of the VP module at increasing pediatric residents’ knowledge with respect to antimicrobial prescribing and AMR; and (b) evaluation of residents’ satisfaction with the VP module as an educational strategy for AMS education. This study was approved by the Research Ethics Board at the IWK Health Centre, Halifax, Canada. Participants provided written informed consent.

#### Phase 1: Virtual patient (VP) module development and validation

The authors designed the VP module using the online platform DecisionSim^TM^, Version 3.2.2, (Kynectiv, Inc., Chadds Ford, PA, USA). A pediatric complicated community acquired pneumonia (CCAP) case was developed using branched design. The six main decision points were based on AMS principles: optimizing diagnosis, antimicrobial selection, dosing, de-escalation, administration route, and duration of treatment [2]. National and international guidelines for management of CCAP as well as local susceptibility patterns for common bacteria (antibiogram) were incorporated into the case. Factors that promote AMR, including inappropriate antimicrobial use, were also presented at the end of the case using text and video clips. Learners received feedback on each decision they made.

The module was evaluated for content validity by 3 infectious diseases physicians and 1 medical microbiologist using a published reviewer checklist for VP design [11]. This questionnaire consisted of 32 questions using a 5 point Likert scale (from strongly disagree to strongly agree) covering authenticity of the patient encounter and consultation, professional approach in the consultation, coaching during consultation, and overall judgment of the case, as well as open-ended questions about strengths and weaknesses. The appropriateness of the case and ease of navigation were evaluated by three pediatric residents from the University of Toronto, Ontario, Canada using a previously published validated online survey [12]. This survey consisted of 12 questions using a 5 point Likert scale (from strongly disagree to strongly agree) covering authenticity of the VP case, engagement with the case, coaching during the case, learning effectiveness of the case (i.e. preparedness to manage a real life case), overall judgment of the case, as well as open-ended questions about strengths and weaknesses [12].

#### Phase 2a: Evaluation of the effectiveness of the VP module

This phase was designed as a pre- and post-intervention evaluation to assess the effect of the VP module on pediatric residents’ knowledge about antimicrobial prescribing and AMR. Potential participants were recruited through email invitation with two follow up email reminders from January to February 2016. A consent form was included in the email invitation. Eligible participants included pediatric residents at all training levels (Postgraduate Year 1 to 4) (N = 24) at the IWK Health Centre.

A previously published validated survey [10], modified to reflect our center’s local antimicrobial susceptibility patterns, was used to measure residents’ mean knowledge scores about AMS principles at three time points: Time point 1: before completing the VP module, Time point 2: immediately after completing the module, and Time point 3: four months after completing the module. The survey included seven “single best answer” multiple choices questions (MCQs). Four MCQs assessed knowledge of AMR and knowledge of local antimicrobial susceptibility patterns of the most common bacteria in pediatric infections; and three MCQs covered application of antimicrobial prescribing principles in pediatric hospital settings (Additional file 1: knowledge MCQs). After completing the pre-test, participants were given access to the online VP module to complete within 1 week. Post-test surveys were sent to participants via email after completing the module. Three MCQs that covered application of antimicrobial prescribing in pediatric hospital settings were changed in the second post-test.

#### Phase 2b: Evaluation of residents’ satisfaction with the VP module

Residents’ satisfaction with the VP modules for antimicrobial education was assessed using the same questionnaire used in the validation phase [12], following completion of the first post-test survey.

#### Statistical analysis

The mean and standard deviation were used to describe knowledge score. Missing scores from post-test (N = 2/9) were replaced by the mean. We transposed the data from wide to long data using the data restructure feature resulting in 9 physicians times 3 repeated measures which equaled 27 data records. Then we conducted Generalized Linear Mixed Repeated Measures (RM) Analysis to assess the effect of the VP module on participants’ knowledge across time. Time point 3 (4 months after completing the module) was used as reference for comparison in the analysis. Independent groups *t* test was used to compare mean knowledge score between postgraduate year (PGY) 1 or 2 residents and PGY 3 or 4 residents. Survey responses regarding residents’ satisfaction with the module were reported as proportions.

### Results

#### Virtual patient (VP) module development and validation

All 4 faculty reviewers agreed that the case represented a typical clinical scenario. Three of 4 reviewers agreed that the case triggered the learners’ clinical reasoning. One reviewer felt that the case encouraged over investigation (Additional file 2: Reviewer checklist). The case was edited based on reviewers’ comments. All 3 residents who piloted the module agreed that it was easy to navigate and reflected a real life case. The key features of the VP module are shown in Table 1.

**Table 1:** Key Features of a Virtual Patient module to educate pediatric residents about principles Antimicrobial Stewardship Practices

Branching design was used
The 6 main decision points were based on AMS principles: 1. optimizing diagnosis, 2. antimicrobial selection, 3. dosing, 4. de-escalation, 5. administration route, and 6. duration of treatment)
Different types of questions (triggers) were used as appropriate. E.g. free text for admission plan, MCQs for antibiotic selection
Media including pictures and videos were used to present clinical and microbiology data
Residents received feedback on each right or wrong decision
Antimicrobial pharmacokinetics and pharmacodynamics were incorporated
Access to different national guidelines were provided at end of the module

#### Effectiveness of the VP module on pediatric residents’ knowledge about antimicrobial prescribing and AMR

Nine of 24 pediatric residents completed the pre-test knowledge survey, of which seven completed the VP module and post intervention knowledge questionnaires. The response rate for completing the pre and post-test phases was 29% (7/24). Of these 7 residents: 3 were in PGY 1–2 while 4 were in PGY 3–4. Overall mean knowledge scores before, immediately after, and 4 months after the module were 58.2%, 66.6%, and 71.6%, respectively. Knowledge scores were significantly lower at time point 1 than time point 3 (p = 0.003), while scores at time points 2 and 3 were similar (p = 0.33) (Table 2).

**Table 2:** Generalized linear repeated measures analysis of the effect of the VP module on participants’ knowledge score across time (N = 27)

	Beta coefficient	Std. Err.	95% C.I (Beta coefficient)		p-value
			Lower	Upper	
(Intercept)	71.66	3.06	65.33	77.99	< 0.001
Time 1 = pre-test	− 13.44	3.98	21.67	5.21	0.003
Time 2 = 1st post-test	− 5.00	5.09	15.52	5.52	0.33
Time 3 = 4 month post-test (reference comparison)	0				

Mean knowledge score by level of training is shown in the Additional file 3: Table S1. There was no significant difference in pre-test knowledge scores between PGY 1–2 and PGY 3–4, p = 0.25.

#### Participants’ satisfaction with the VP modules for AMS education

Seven participants completed the VP module and the satisfaction survey. Satisfaction with the VP module was high overall (i.e. all agreed or strongly agreed) in terms of its simulation of real life, engagement with the case, feedback on the participant’s decisions, and perceived preparedness for managing a similar real life case (Table 3).

**Table 3:** Residents’ Satisfaction Survey (N = 7)

Question	Strongly disagree	Disagree	Neutral	Agree	Strongly agree
I felt I had to make the same decisions a doctor would make in real life	0 (0%)	0 (0%)	0 (0%)	2 (28.5%)	5 (71.4%)
I felt I were the doctor caring for this patient	0 (0%)	0 (0%)	0 (0%)	5 (71.4%)	2 (28.5%)
I was actively engaged in revising my initial image of the patient’s problem as new information became available	0 (0%)	0 (0%)	0 (0%)	3 (42.8%)	4 (57.1%)
I was actively engaged in creating a short summary of the patient’s problem using medical terms	0 (0%)	0 (0%)	2 (28.5%)	3 (42.8%)	2 (28.5%)
I was actively engaged in thinking about which findings supported or refuted each diagnosis in my differential diagnosis	0 (0%)	0 (0%)	0 (0%)	3 (42.8%)	4 (57.1%)
The questions I was asked were helpful in enhancing my clinical reasoning (collecting, analyzing, interpreting information and making decisions) in this case	0 (0%)	0 (0%)	0 (0%)	3 (42.8%)	4 (57.1%)
The feedback I received was helpful in enhancing my decision making in this module	0 (0%)	0 (0%)	0 (0%)	3 (42.8%)	4 (57.1%)
After completing this case I feel better prepared to care for a real life patient to practice antimicrobial prescribing principles	0 (0%)	0 (0%)	0 (0%)	3 (42.8%)	4 (57.1%)
Overall, working through this case was a worthwhile learning experience	0 (0%)	0 (0%)	0 (0%)	3 (42.8%)	4 (57.1%)

### Discussion

The development and validation phase showed that the online VP module simulated real life cases and enhanced learning of AMS principles and AMR among pediatric residents. VP modules may also enhance clinical reasoning skills in antimicrobial prescribing as shown in the validation phase. Pediatric residents’ knowledge about principles of antimicrobial prescribing and AMR appeared to increase after completing a VP module that incorporates AMS principles.

Based on our literature review, this is one of first studies to assess the effectiveness of online VP modules in postgraduate AMS education. Most prior studies reporting on AMS instructional methods assessed passive learning methods (e.g. group presentations and guideline dissemination) [13, 14]. Such methods are unlikely to result in sustained behavioral improvement in antimicrobial prescribing [2].

VP modules have been used mainly in undergraduate medical education and there is growing interest in post-graduate teaching [6]. Cook et al. has published a systemic review and meta-analysis on the use of computerized VP modules in health professions education that showed improvement in knowledge and clinical reasoning skills when VP is compared with no intervention or non-computer instruction. However there was considerable heterogeneity in study design and populations included in these studies [6]. A recent study evaluated the use of online learning in AMS education among 606 medical students. Six online modules were developed covering different AMS topics. The modules included both theoretical information as well as virtual patients. Significant improvement in mean knowledge scores was observed between pre and post intervention assessments (5.78 vs 8.45, p < 0.001) [15]. Similarly, Heath et al. reported an improvement in AMS knowledge among 103 nurses at long-term care facilities after completing online learning modules. Pre and post knowledge scores were 75% and 86%, respectively (p < 0.001). However, details of the instructional methods used were not described [16].

Resident physicians expressed a high level of satisfaction with the VP for AMS education. All participants agreed that the module simulated real-life cases that they might encounter in their daily practice. They also reported that it stimulated their clinical reasoning skills and prepared them to manage a similar case. These findings are in agreement with most studies that evaluated online VP modules [6].

In conclusion, VP modules present an innovate approach to AMS education among resident physicians that merits further study.

## Limitations

This study was a pilot research project and was limited to one module and a small sample of participants, which limits the generalizability of the findings. In addition, we could not incorporate other features in the VP module such as tracking respondents’ time due to limited funding. We were also unable to measure how completion of the VP module affected residents’ antimicrobial prescribing behavior.

## Supplementary information

**Additional file 1.** Antimicrobial Stewardship (AMS) Knowledge Questions.

**Additional file 2.** Reviewer checklist.

**Additional file 3.** Descriptive statistics of residents’ knowledge score Pre- and Post VP module.

## Data Availability

All data generated or analyzed during this study are included in this published article [and its additional information files]. Figure of example of branching node in the VP module is available upon request from the corresponding author.

## Abbreviations

ASP: Antimicrobial Stewardship Programs
AMR: Antimicrobial resistance
VP: Virtual patient
CAP: Community acquired pneumonia
CCAP: Complicated community acquired pneumonia
PGY: Postgraduate year
