# Supplementary material for: On-line virtual patient learning: a pilot study of a new modality in antimicrobial stewardship education for pediatric residents
Source: BMC Res Notes. 2020 Jul 14;13:339. doi: 10.1186/s13104-020-05170-7 (PMC7362648; doi:10.1186/s13104-020-05170-7)
Supplement: Supplementary file 1 — Additional file 1. Antimicrobial Stewardship (AMS) Knowledge Questions. [file 13104_2020_5170_MOESM1_ESM.pdf]

Antimicrobial Stewardship (AMS) Knowledge Questions

12. For each of the following pairs of prescriptions, which contributes MOST to promoting antimicrobial resistance?

Circle only one of each pair. *(Correct answers are underlined)*

a) **Lower doses** **or** Higher doses

Lower doses permit the bacteria that have low level resistance to multiply and increase the chances that they will in turn be resistant.

b) **Longer courses** **or** Shorter courses (≤ 7 days)

Longer courses expose the bacteria in the body to antibiotics for longer periods of time thereby promoting the survival of more resistant bacteria.

c) Ampicillin **or** **Piperacillin-tazobactam** (for equivalent time)

Since piperacillin- tazobactam is broader spectrum, it will eradicate many more susceptible bacteria and leave only resistant ones which are then left at the end of therapy to multiply.

d) Gentamicin **or** **Ceftriaxone** (for equivalent time)

Ceftriaxone has a broader spectrum of activity and selects for more resistant bacteria including *Clostridium difficile.*

e) **Azithromycin** **or** Clarithromycin (for equivalent time)

Azithromycin has a very long half-life of 5 days. It will be present for a prolonged period of time at low levels thus selecting for macrolide resistant bacteria.

13. How many days of antibiotics does it take to change the flora (bacteria) in the gut and pharynx to more resistant bacteria?

Please check only one.

_ **3 days**
_ 6 days
_ 9 days

When systemic antibiotics are used, the potential for changing the bacteria in the oropharynx and the gut is large. Thus, resistant bacteria can emerge while the patient is being treated for infection. The longer a patient receives antibiotics, the greater potential that the bacteria will change. No one knows for sure in all cases, but the studies have shown that this occurs fairly early (~3 days) after starting antimicrobials. Thus, to avoid the emergence of resistance, discontinuing antibiotics is paramount if there is no infection and to use the shortest duration that is recommended when infection is present.

14. According to the Capital Health (CDHA) 2014 antibiogram, what is the % of susceptibility that was found for the following bacteria?

The **antibiogram is found on the microbiology site on chda.nshealth.ca.**
(http://www.cdha.nshealth.ca/pathology-laboratory-medicine/microbiology-services-diagnostic/bacteriology)
It is a useful measure of the antibiotic resistance profiles of commonly isolated bacteria in the region and is produced yearly by the Microbiology Laboratory at CDHA (now Nova Scotia Health Authority). Its limitation for pediatric use is that it is based upon susceptibility patterns from different age groups (i.e., including adults) across the former CDHA area.

a) Streptococcus pneumonia susceptible to penicillin (Non cerebrospinal fluid (CSF) samples)
☐ 20% ☐ 40% ☐ 50% **☐ 70%** ☐ 90%

In the 2014 antibiogram around 70 % of pneumococcal isolates are susceptible to penicillin (non- CSF samples).

b) Group A *Streptococcus* susceptible to erythromycin
☐50% ☐ 60% ☐70% **☐80%** ☐100%

The 2014 antibiogram indicated 80% erythromycin susceptibility among GAS isolates.

c) Methicillin-resistant Staphylococcus aureus (MRSA) susceptible to clindamycin
☐10% ☐ 20% **☐40%** ☐60% ☐ 80%

The percentage of MRSA susceptible to clindamycin is 40%.

15. Which one of the following antibiotics would be LEAST likely to increase the risk of developing of *Clostridium difficile* infection?

- cefotaxime
- clindamycin
- piperacillin
- vancomycin
- **gentamicin**

Although any antimicrobial can increase the risk of *C. difficile*, within this list, gentamicin has the narrowest spectrum of activity and is the agent that least likely to increase the risk for *Clostridium difficile*.

The following are scenarios about principles of antimicrobial stewardship.

16. You are admitting a previously healthy 3 year old child who has received all recommended Immunizations according to NS childhood immunization schedule. The child had cough, fever and chest pain for 4 days. He has a temperature of 38.5°C, a respiratory rate of 30/min, a heart rate of 90/minute and an oxygen saturation of 98% on room air. His radiograph shows a moderate size right middle lobe (RML) infiltrate. What would be the recommended empiric antimicrobial therapy?

_ cefuroxime
_ **ampicillin**
_ cefuroxime and clindamycin
_ Ceftriaxone

The Canadian and AAP pneumonia guidelines recommend intravenous ampicillin for a child who is admitted with an uncomplicated pneumonia AND who is not severely ill. This is appropriate since it covers the most common organism likely to be causing the pneumonia (*Streptococcus pneumonia*).

17. The child shows clinical improvement (afebrile after 48 hours and eating) and the blood cultures are negative. What would you likely prescribe as outpatient oral antimicrobial therapy?

_ cefuroxime axetil
_ **amoxicillin**
_ Clavulin® (amoxillin and clavulanate)

Amoxicillin is the drug of choice for step-down therapy.

18. What is the recommended total length of antibiotic therapy (IV and oral) assuming no complications?

_ 5 days
_ 7 days
_ **10 days**

The Canadian Pediatrics Society (CPS) and Infecttious Diseases Society of America (IDSA) recommend 10 day therapy for uncomplicated pneumonia. The length of therapy also depends on the rapidity of clinical response.

Four months survey clinical scenarios:

16. A 6 year-old boy presents with redness and swelling of the left foot. No history of fever. On examination, he is well looking, temperature 37.3 °C. Examination of the left foot showed small splinter on the dorsal aspect of the foot distal to the ankle, diffuse soft tissue edema, redness and warmth near the ankle, normal range of motion of left ankle with no fluctuation, joint effusion or bony tenderness.
How would you manage this patient?

_ Incision and drainage
 Oral antibiotics
_ IV antibiotics
_ Topical mupirocin
_ No treatment

Outpatient therapy is recommended for patients with cellulitis who do not have systemic manifestation, altered mental status, or hemodynamic instability.

17. Which antibiotics would you prescribe assuming he has no allergies?
_ Clindamycin
_ Erythromycin
_ Penicillin V
 Cephalexin

Cellulitis is usually caused by methicillin susceptible staphylococci (MSSA) and group A β-hemolytic streptococcus So, first generation cephalosporin is the narrowest spectrum and best choice to cover both.

18. What is the recommended total length of antibiotic therapy assuming no complications?
_ 3 days
 5 days
_ 10 days

In cases of uncomplicated cellulitis, a 5-day course of antimicrobial therapy is as effective as a 10-day course, if clinical improvement has occurred by 5 days.
