# Supplementary material for: On-line virtual patient learning: a pilot study of a new modality in antimicrobial stewardship education for pediatric residents
Source: BMC Res Notes. 2020 Jul 14;13:339. doi: 10.1186/s13104-020-05170-7 (PMC7362648; doi:10.1186/s13104-020-05170-7)
Supplement: Supplementary file 2 — Additional file 2. Reviewer checklist. [file 13104_2020_5170_MOESM2_ESM.pdf]

Reviewer Checklist

Lists all the questions in the survey and displays a summary with detailed statistics and a chart for each question. Free text responses are not included.

Table of contents

Report info....1

Question 1: This case is relevant for real medical practice....2

Question 2: This case gives a typical presentation of a patient with this disease....3

Question 3: The media (pictures, audio files, videos, etc) support the realism in this case....4

Question 4: The cognitive tasks students complete during the case work-up correspond to real-life phy....5

Question 5: The numbers of decisions students make in this case correspond to real life decisions....6

Question 6: The chunks of information presented in this case reflect the quantity of information the 7

Question 7: The case triggers the user to actively gather information necessary for diagnosis and the....8

Question 8: The case triggers the user (by e.g., prompts and feedback) to summarize the clinical prob....9

Question 9: The case triggers the user (by e.g., prompts and/or reference material) to interpret the 10

Question 10: The case triggers the user (by e.g., prompts and feedback) to iteratively re-evaluate th....11

Question 11: The case triggers the user (by e.g., prompts and feedback) to infer consequences of the 12

Question 12: The case triggers the user (by e.g., prompts and feedback) to differentiate between impo....13

Question 13: The case triggers the users (by e.g., prompts and feedback) to differentiate features as....14

Question 14: The case triggers the user (by e.g., prompts) to generate hypothesis early in the diagno....15

Question 15: The case triggers the user (by e.g., prompts, advance organizers) to link the case with 16

Question 16: The degree of difficulty of the case is appropriate for the target group....17

Question 17: Media (pictures, video, audio, diagrams, graphics) are used, whenever superior to verbal....18

Question 18: The case helps the user to interpret pathological data in an authentic format, by offeri....19

Question 19: The case triggers the user at the end of the case (by e.g., prompts) to point out the mo....20

Question 20: The amount of information presented simultaneously (the so-called cognitive load) is app....21

Question 21: The case uses attributes (e.g., highlighting via bold or colour, pointers) to point out 22

Question 22: The case gives users feedback on all decisions they take....23

Question 23: The feedback in the case is elaborated by explaining why something is right....24

Question 24: The feedback in the case is elaborated by explaining why something is wrong....25

Question 25: The case offers possibilities for self-assessment....26

Question 26: The case triggers the user (by e.g., prompts, feedback) to evaluate their actions of the....27

Question 27: The case triggers the user (by. e.g., prompts, feedback) to evaluate their diagnostic re....28

Question 28: The case triggers the user (by. e.g., prompts, feedback) to evaluate their diagnostic re....29

Question 29: Overall, this case is very well suited to enhance learning in the target group....30

Question 30: Overall, this case is very well suited to foster clinical reasoning in the target group....31

Question 31: Special weaknesses of this case....32

Question 32: Special strengths of this case....33

Report info

**Report date:**	Monday, January 18, 2016 7:03:14 PM AST
**Start date:**	Friday, December 11, 2015 12:27:00 AM AST
**Stop date:**	Sunday, January 31, 2016 12:27:00 AM AST
**Stored responses:**	5
**Number of completed responses:**	4
**Number of invitees:**	4
**Invitees that responded:**	4
**Invitee response rate:**	100%

Question 1

This case is relevant for real medical practice.

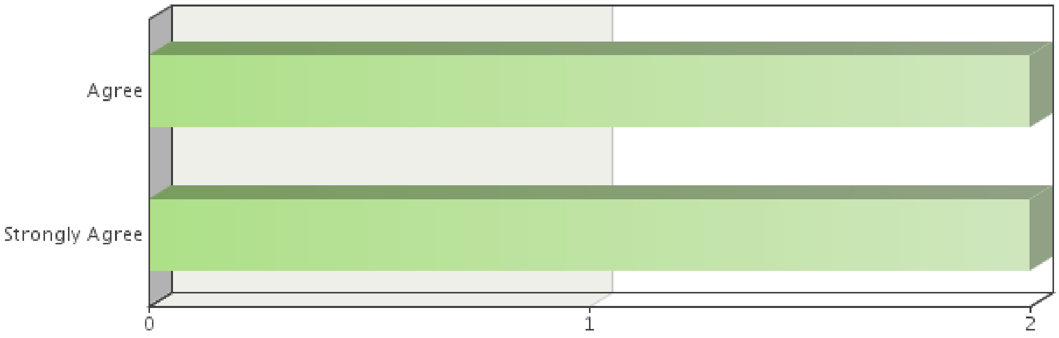

Frequency table

Levels	Absolute frequency	Cum. absolute frequency	Relative frequency	Cum. relative frequency	Adjusted relative frequency	Cum. adjusted relative frequency
Agree	2	2	40%	40%	50%	50%
Strongly Agree	2	4	40%	80%	50%	100%
Sum:	4	-	80%	-	100%	-
Not answered:	1	-	20%	-	-	-

Average:	4.5	Minimum:	4	Variance:	0.33
Median:	4.5	Maximum:	5	Std. deviation:	0.58

Total answered: 4

Question 2

This case gives a typical presentation of a patient with this disease.

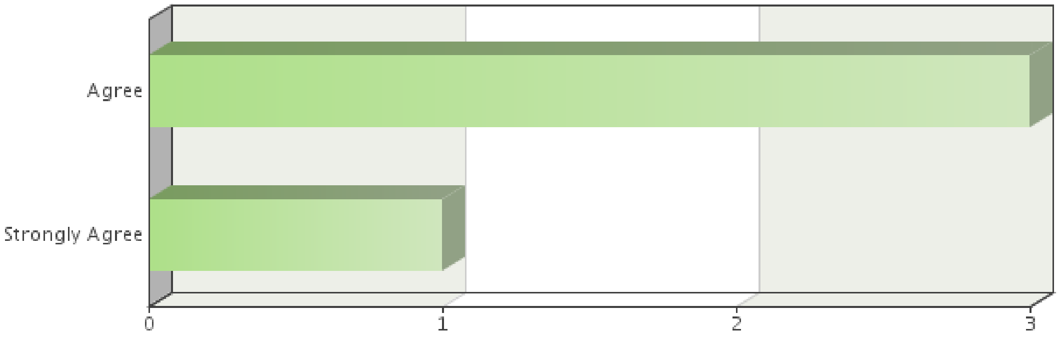

Frequency table

Levels	Absolute frequency	Cum. absolute frequency	Relative frequency	Cum. relative frequency	Adjusted relative frequency	Cum. adjusted relative frequency
Agree	3	3	60%	60%	75%	75%
Strongly Agree	1	4	20%	80%	25%	100%
Sum:	4	-	80%	-	100%	-
Not answered:	1	-	20%	-	-	-

Average:	4.25	Minimum:	4	Variance:	0.25
Median:	4	Maximum:	5	Std. deviation:	0.5

Total answered: 4

Question 3

The media (pictures, audio files, videos, etc) support the realism in this case.

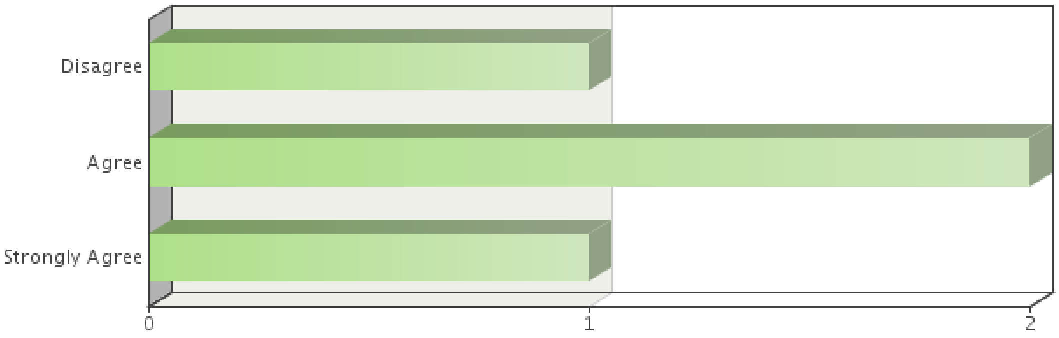

Frequency table

Levels	Absolute frequency	Cum. absolute frequency	Relative frequency	Cum. relative frequency	Adjusted relative frequency	Cum. adjusted relative frequency
Disagree	1	1	20%	20%	25%	25%
Agree	2	3	40%	60%	50%	75%
Strongly Agree	1	4	20%	80%	25%	100%
Sum:	4	-	80%	-	100%	-
Not answered:	1	-	20%	-	-	-

Average:	3.75	Minimum:	2	Variance:	1.58
Median:	4	Maximum:	5	Std. deviation:	1.26

Total answered: 4

Question 4

The cognitive tasks students complete during the case work-up correspond to real-life physician tasks.

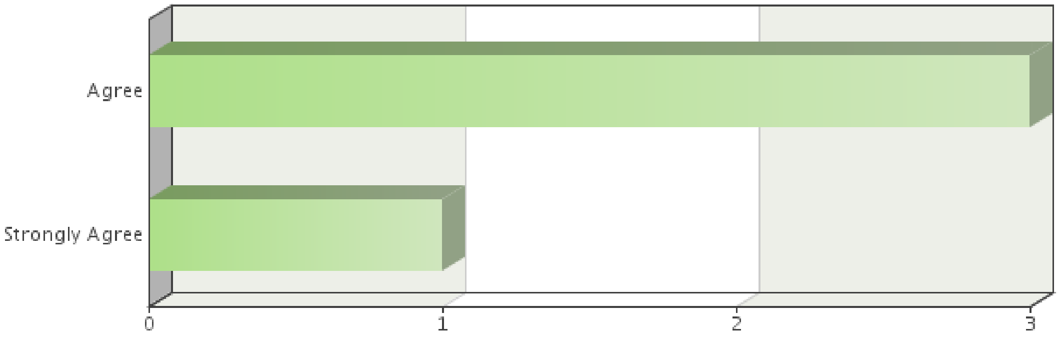

Frequency table

Levels	Absolute frequency	Cum. absolute frequency	Relative frequency	Cum. relative frequency	Adjusted relative frequency	Cum. adjusted relative frequency
Agree	3	3	60%	60%	75%	75%
Strongly Agree	1	4	20%	80%	25%	100%
Sum:	4	-	80%	-	100%	-
Not answered:	1	-	20%	-	-	-

Average:	4.25	Minimum:	4	Variance:	0.25
Median:	4	Maximum:	5	Std. deviation:	0.5

Total answered: 4

Question 5

The numbers of decisions students make in this case correspond to real life decisions.

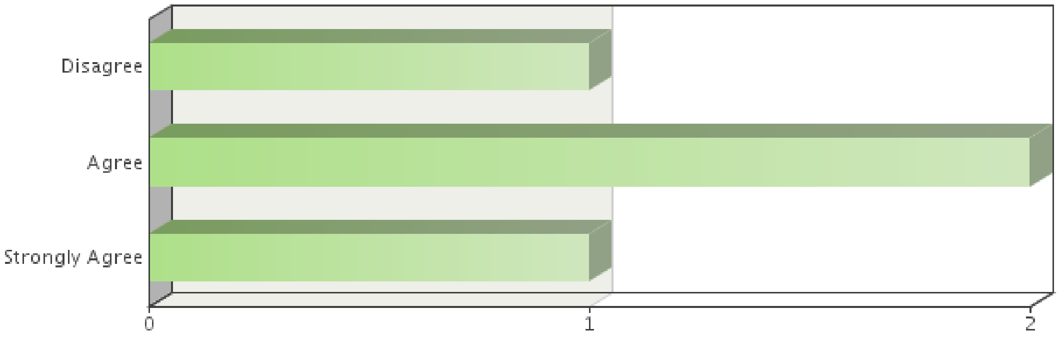

Frequency table

Levels		Absolute frequency	Cum. absolute frequency	Relative frequency	Cum. relative frequency	Adjusted relative frequency	Cum. adjusted relative frequency
Disagree		1	1	20%	20%	25%	25%
Agree		2	3	40%	60%	50%	75%
Strongly Agree		1	4	20%	80%	25%	100%
Sum:		4	-	80%	-	100%	-
Not answered:		1	-	20%	-	-	-

Average:	3.75	Minimum:	2	Variance:	1.58
Median:	4	Maximum:	5	Std. deviation:	1.26

Total answered: 4

Question 6

The chunks of information presented in this case reflect the quantity of information the user will be confronted with in real practice.

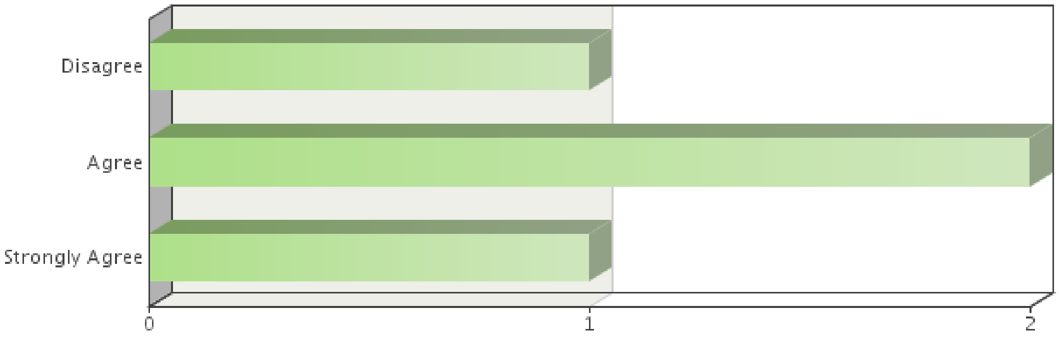

Frequency table

Levels	Absolute frequency	Cum. absolute frequency	Relative frequency	Cum. relative frequency	Adjusted relative frequency	Cum. adjusted relative frequency
Disagree	1	1	20%	20%	25%	25%
Agree	2	3	40%	60%	50%	75%
Strongly Agree	1	4	20%	80%	25%	100%
Sum:	4	-	80%	-	100%	-
Not answered:	1	-	20%	-	-	-

Average:	3.75	Minimum:	2	Variance:	1.58
Median:	4	Maximum:	5	Std. deviation:	1.26

Total answered: 4

Question 7

The case triggers the user to actively gather information necessary for diagnosis and therapy (e.g., history questions to ask, physical exams to perform, labs and diagnostic tests to order).

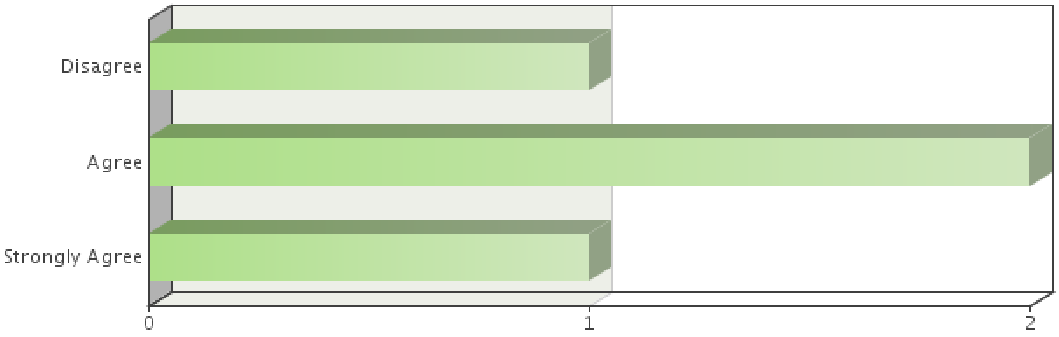

Frequency table

Levels	Absolute frequency	Cum. absolute frequency	Relative frequency	Cum. relative frequency	Adjusted relative frequency	Cum. adjusted relative frequency
Disagree	1	1	20%	20%	25%	25%
Agree	2	3	40%	60%	50%	75%
Strongly Agree	1	4	20%	80%	25%	100%
Sum:	4	-	80%	-	100%	-
Not answered:	1	-	20%	-	-	-

Average:	3.75	Minimum:	2	Variance:	1.58
Median:	4	Maximum:	5	Std. deviation:	1.26

Total answered: 4

Question 8

The case triggers the user (by e.g., prompts and feedback) to summarize the clinical problem in professional medical terms shortly.

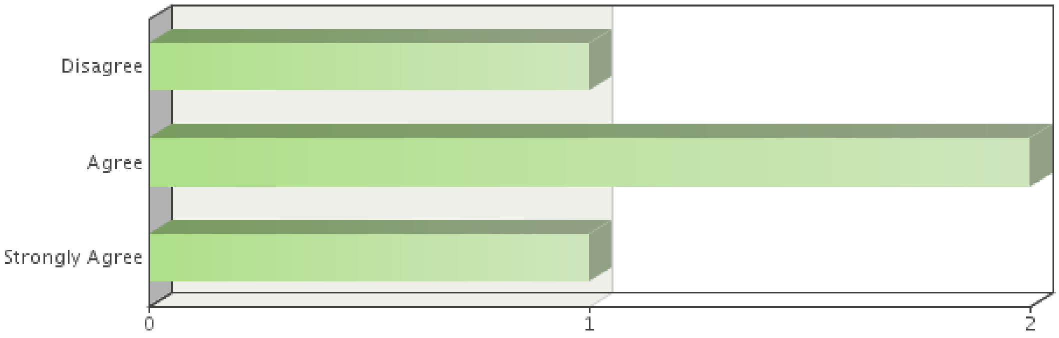

Frequency table

Levels			Absolute frequency	Cum. absolute frequency	Relative frequency	Cum. relative frequency	Adjusted relative frequency	Cum. adjusted relative frequency
Disagree			1	1	20%	20%	25%	25%
Agree			2	3	40%	60%	50%	75%
Strongly Agree			1	4	20%	80%	25%	100%
Sum:			4	-	80%	-	100%	-
Not answered:			1	-	20%	-	-	-
Average:	3.75	Minimum:	2		Variance:		1.58	
Median:	4	Maximum:	5		Std. deviation:		1.26	

Total answered: 4

Question 9

The case triggers the user (by e.g., prompts and/or reference material) to interpret the data presented critically.

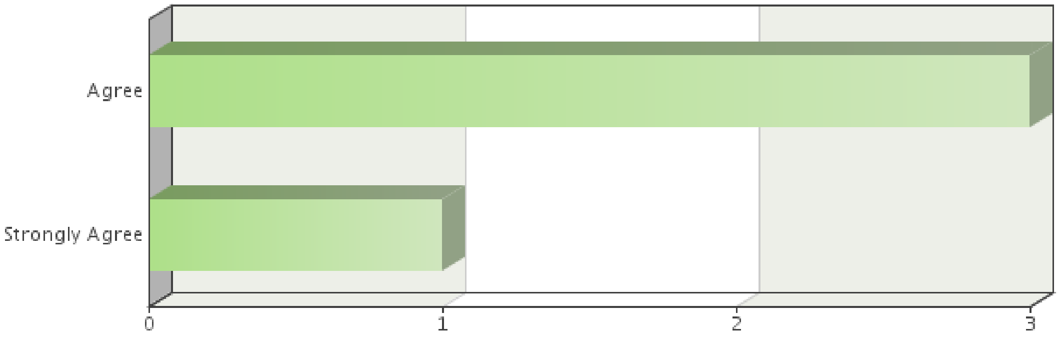

Frequency table

Levels		Absolute frequency	Cum. absolute frequency	Relative frequency	Cum. relative frequency	Adjusted relative frequency	Cum. adjusted relative frequency
Agree		3	3	60%	60%	75%	75%
Strongly Agree		1	4	20%	80%	25%	100%
Sum:		4	-	80%	-	100%	-
Not answered:		1	-	20%	-	-	-
Average:	4.25	Minimum:	4	Variance:	0.25		
Median:	4	Maximum:	5	Std. deviation:	0.5		

Total answered: 4

Question 10

The case triggers the user (by e.g., prompts and feedback) to iteratively re-evaluate the suspected diagnoses in light of newly gained information.

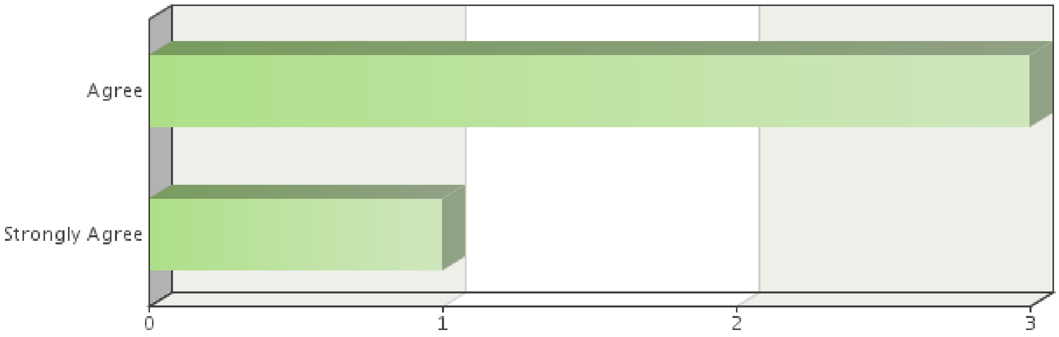

Frequency table

Levels				Absolute frequency	Cum. absolute frequency	Relative frequency	Cum. relative frequency	Adjusted relative frequency	Cum. adjusted relative frequency
Agree				3	3	60%	60%	75%	75%
Strongly Agree				1	4	20%	80%	25%	100%
Sum:				4	-	80%	-	100%	-
Not answered:				1	-	20%	-	-	-
Average:	4.25	Minimum:		4		Variance:		0.25	
Median:	4	Maximum:		5		Std. deviation:		0.5	

Total answered: 4

Question 11

The case triggers the user (by e.g., prompts and feedback) to infer consequences of the findings for diagnosis and therapy.

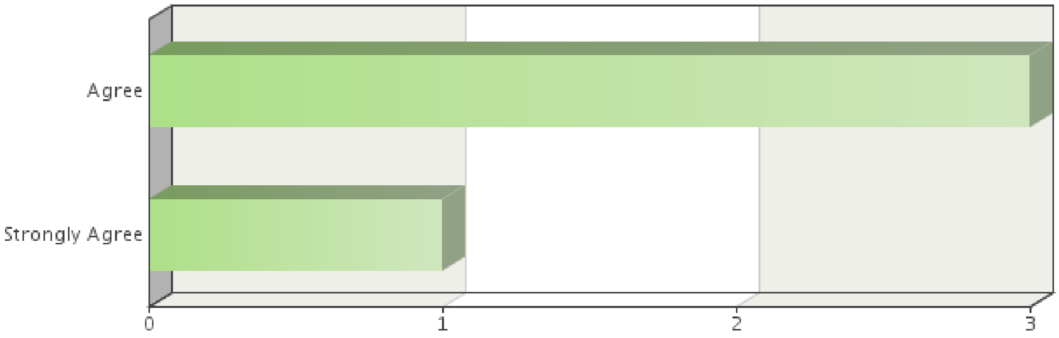

Frequency table

Levels			Absolute frequency	Cum. absolute frequency	Relative frequency	Cum. relative frequency	Adjusted relative frequency	Cum. adjusted relative frequency
Agree			3	3	60%	60%	75%	75%
Strongly Agree			1	4	20%	80%	25%	100%
Sum:			4	-	80%	-	100%	-
Not answered:			1	-	20%	-	-	-
Average:	4.25	Minimum:		4		Variance:	0.25	
Median:	4	Maximum:		5		Std. deviation:	0.5	

Total answered: 4

Question 12

The case triggers the user (by e.g., prompts and feedback) to differentiate between important and less important information.

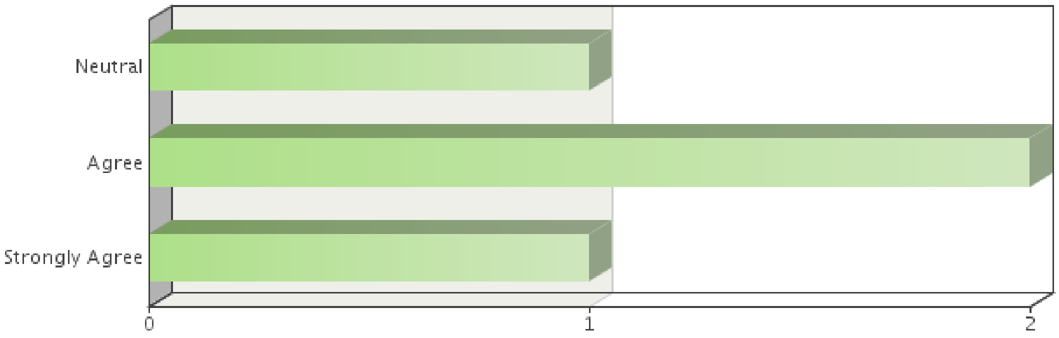

Frequency table

Levels				Absolute frequency	Cum. absolute frequency	Relative frequency	Cum. relative frequency	Adjusted relative frequency	Cum. adjusted relative frequency
Neutral				1	1	20%	20%	25%	25%
Agree				2	3	40%	60%	50%	75%
Strongly Agree				1	4	20%	80%	25%	100%
Sum:				4	-	80%	-	100%	-
Not answered:				1	-	20%	-	-	-
Average:	4	Minimum:			3	Variance:		0.67	
Median:	4	Maximum:			5	Std. deviation:		0.82	

Total answered: 4

Question 13

The case triggers the users (by e.g., prompts and feedback) to differentiate features as either “discriminating” or “confining” for differential diagnoses.

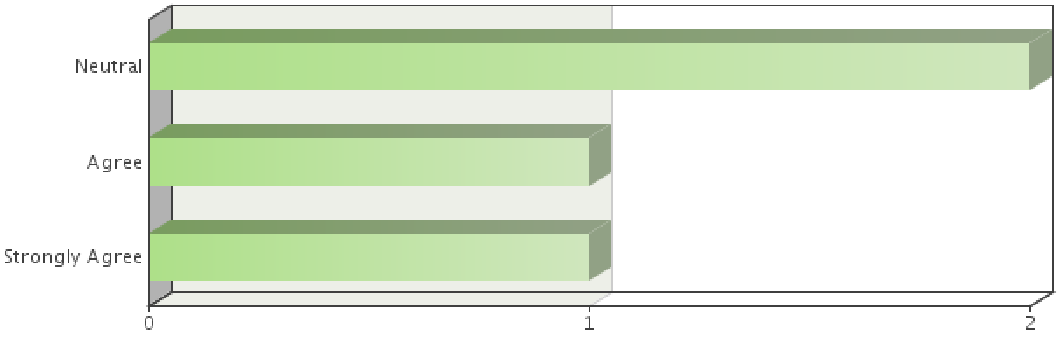

Frequency table

Levels	Absolute frequency	Cum. absolute frequency	Relative frequency	Cum. relative frequency	Adjusted relative frequency	Cum. adjusted relative frequency
Neutral	2	2	40%	40%	50%	50%
Agree	1	3	20%	60%	25%	75%
Strongly Agree	1	4	20%	80%	25%	100%
Sum:	4	-	80%	-	100%	-
Not answered:	1	-	20%	-	-	-

Average:	3.75	Minimum:	3	Variance:	0.92
Median:	3.5	Maximum:	5	Std. deviation:	0.96

Total answered: 4

Question 14

The case triggers the user (by e.g., prompts) to generate hypothesis early in the diagnostic process.

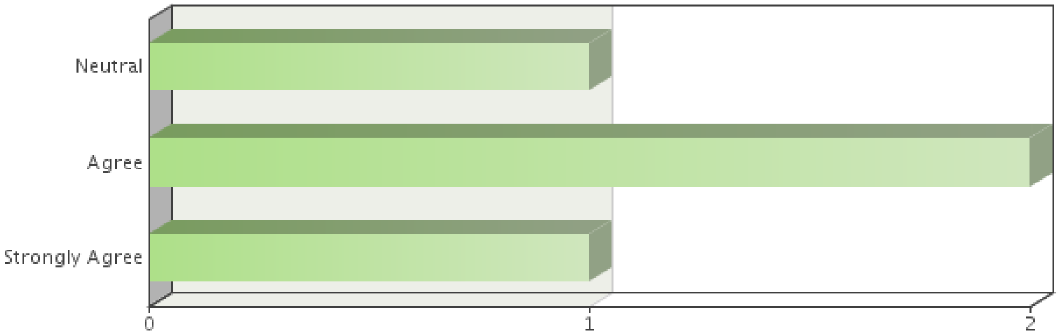

Frequency table

Levels			Absolute frequency	Cum. absolute frequency	Relative frequency	Cum. relative frequency	Adjusted relative frequency	Cum. adjusted relative frequency
Neutral			1	1	20%	20%	25%	25%
Agree			2	3	40%	60%	50%	75%
Strongly Agree			1	4	20%	80%	25%	100%
Sum:			4	-	80%	-	100%	-
Not answered:			1	-	20%	-	-	-
Average:	4	Minimum:		3	Variance:		0.67	
Median:	4	Maximum:		5	Std. deviation:		0.82	

Total answered: 4

Question 15

The case triggers the user (by e.g., prompts, advance organizers) to link the case with their prior knowledge.

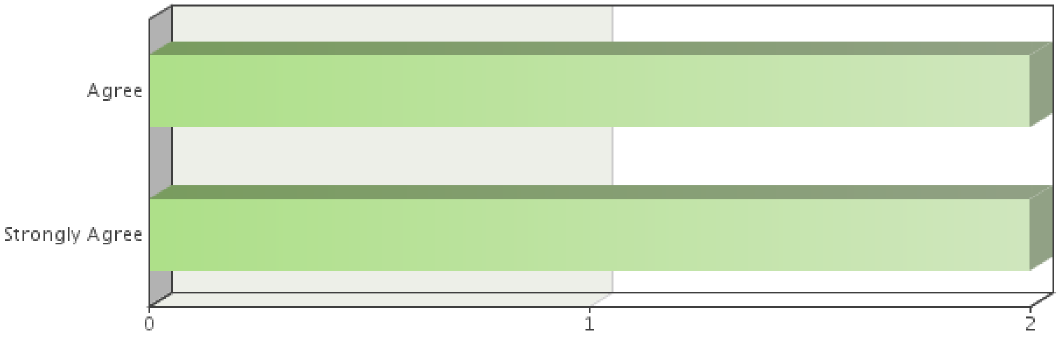

Frequency table

Levels		Absolute frequency	Cum. absolute frequency	Relative frequency	Cum. relative frequency	Adjusted relative frequency	Cum. adjusted relative frequency
Agree		2	2	40%	40%	50%	50%
Strongly Agree		2	4	40%	80%	50%	100%
Sum:		4	-	80%	-	100%	-
Not answered:		1	-	20%	-	-	-
Average:	4.5	Minimum:	4	Variance:	0.33		
Median:	4.5	Maximum:	5	Std. deviation:	0.58		

Total answered: 4

Question 16

The degree of difficulty of the case is appropriate for the target group.

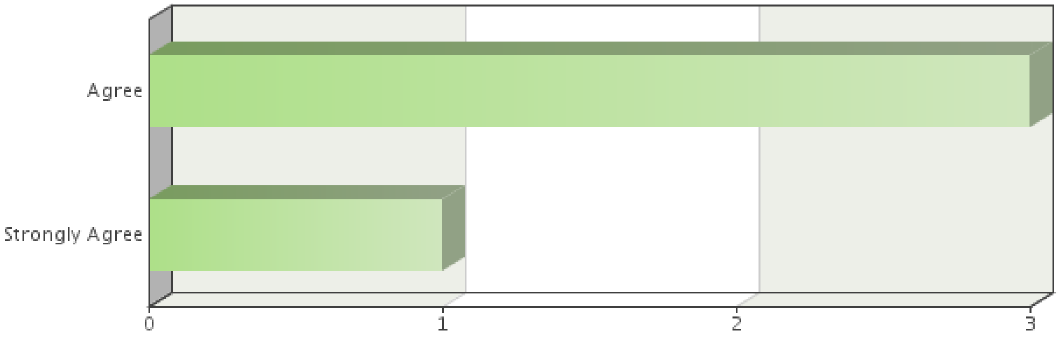

Frequency table

Levels		Absolute frequency	Cum. absolute frequency	Relative frequency	Cum. relative frequency	Adjusted relative frequency	Cum. adjusted relative frequency
Agree		3	3	60%	60%	75%	75%
Strongly Agree		1	4	20%	80%	25%	100%
Sum:		4	-	80%	-	100%	-
Not answered:		1	-	20%	-	-	-

Average:	4.25	Minimum:	4	Variance:	0.25
Median:	4	Maximum:	5	Std. deviation:	0.5

Total answered: 4

Question 17

Media (pictures, video, audio, diagrams, graphics) are used, whenever superior to verbal explanations.

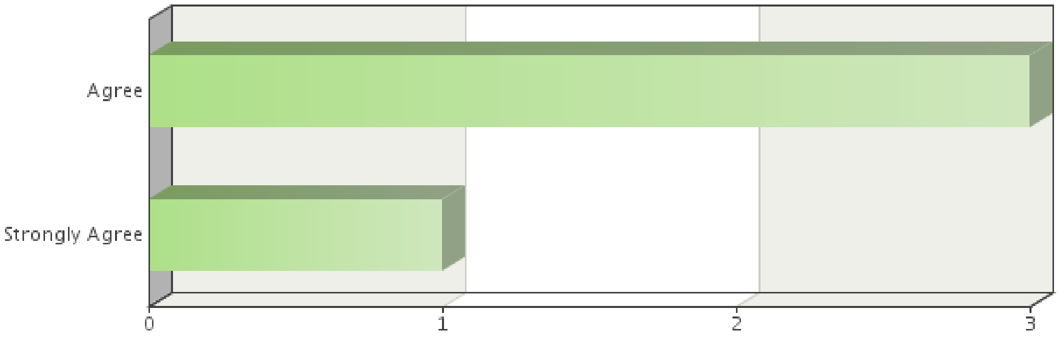

Frequency table

Levels	Absolute frequency	Cum. absolute frequency	Relative frequency	Cum. relative frequency	Adjusted relative frequency	Cum. adjusted relative frequency
Agree	3	3	60%	60%	75%	75%
Strongly Agree	1	4	20%	80%	25%	100%
Sum:	4	-	80%	-	100%	-
Not answered:	1	-	20%	-	-	-

Average:	4.25	Minimum:	4	Variance:	0.25
Median:	4	Maximum:	5	Std. deviation:	0.5

Total answered: 4

Question 18

The case helps the user to interpret pathological data in an authentic format, by offering a normal finding as reference (e.g., pathologic and normal x-ray, sounds, etc).

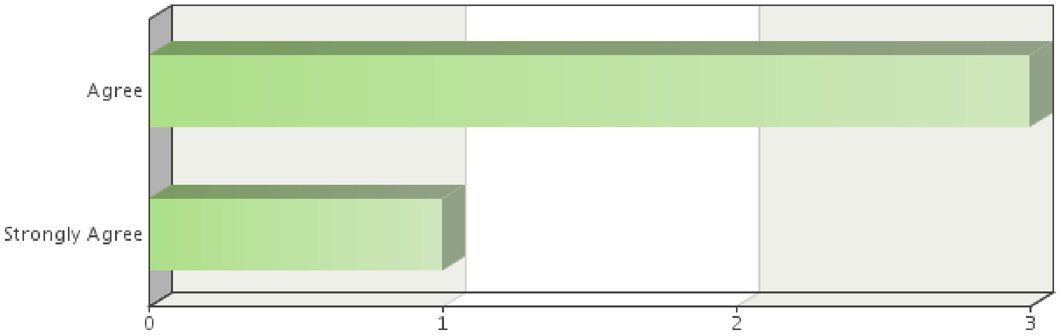

Frequency table

Levels				Absolute frequency	Cum. absolute frequency	Relative frequency	Cum. relative frequency	Adjusted relative frequency	Cum. adjusted relative frequency
Agree				3	3	60%	60%	75%	75%
Strongly Agree				1	4	20%	80%	25%	100%
Sum:				4	-	80%	-	100%	-
Not answered:				1	-	20%	-	-	-
Average:	4.25	Minimum:		4		Variance:		0.25	
Median:	4	Maximum:		5		Std. deviation:		0.5	

Total answered: 4

Question 19

The case triggers the user at the end of the case (by e.g., prompts) to point out the most important information.

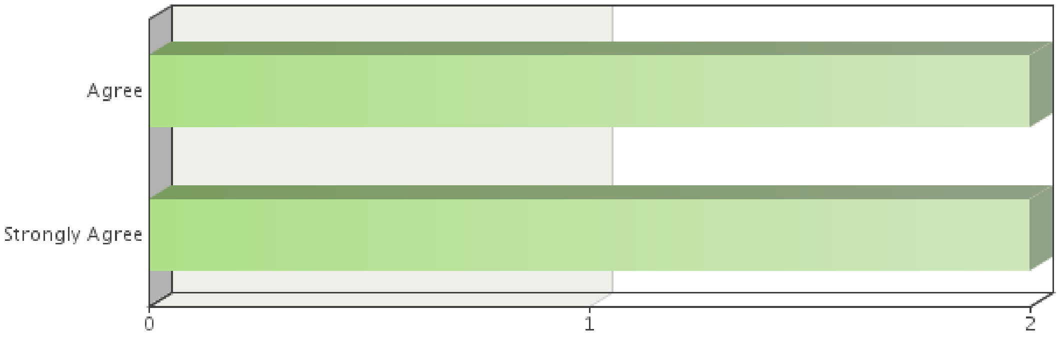

Frequency table

Levels	Absolute frequency	Cum. absolute frequency	Relative frequency	Cum. relative frequency	Adjusted relative frequency	Cum. adjusted relative frequency
Agree	2	2	40%	40%	50%	50%
Strongly Agree	2	4	40%	80%	50%	100%
Sum:	4	-	80%	-	100%	-
Not answered:	1	-	20%	-	-	-

Average:	4.5	Minimum:	4	Variance:	0.33
Median:	4.5	Maximum:	5	Std. deviation:	0.58

Total answered: 4

Question 20

The amount of information presented simultaneously (the so-called cognitive load) is appropriate.

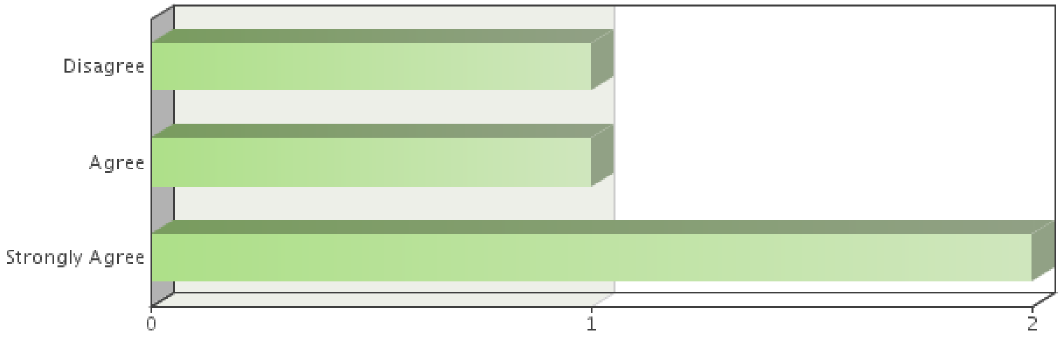

Frequency table

Levels	Absolute frequency	Cum. absolute frequency	Relative frequency	Cum. relative frequency	Adjusted relative frequency	Cum. adjusted relative frequency
Disagree	1	1	20%	20%	25%	25%
Agree	1	2	20%	40%	25%	50%
Strongly Agree	2	4	40%	80%	50%	100%
Sum:	4	-	80%	-	100%	-
Not answered:	1	-	20%	-	-	-

Average:	4	Minimum:	2	Variance:	2
Median:	4.5	Maximum:	5	Std. deviation:	1.41

Total answered: 4

Question 21

The case uses attributes (e.g., highlighting via bold or colour, pointers) to point out the most important information.

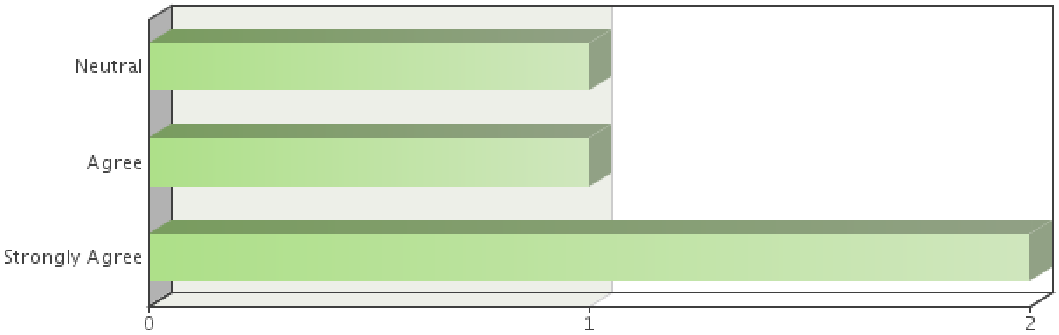

Frequency table

Levels	Absolute frequency	Cum. absolute frequency	Relative frequency	Cum. relative frequency	Adjusted relative frequency	Cum. adjusted relative frequency
Neutral	1	1	20%	20%	25%	25%
Agree	1	2	20%	40%	25%	50%
Strongly Agree	2	4	40%	80%	50%	100%
Sum:	4	-	80%	-	100%	-
Not answered:	1	-	20%	-	-	-

Average:	4.25	Minimum:	3	Variance:	0.92
Median:	4.5	Maximum:	5	Std. deviation:	0.96

Total answered: 4

Question 22

The case gives users feedback on all decisions they take.

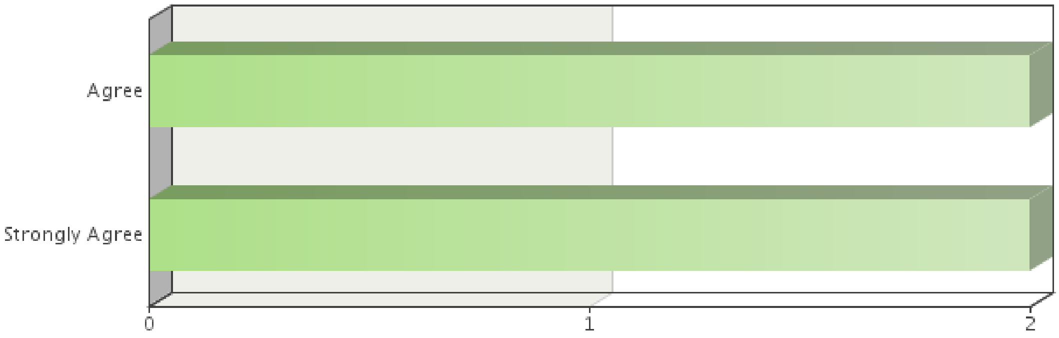

Frequency table

Levels			Absolute frequency	Cum. absolute frequency	Relative frequency	Cum. relative frequency	Adjusted relative frequency	Cum. adjusted relative frequency
Agree			2	2	40%	40%	50%	50%
Strongly Agree			2	4	40%	80%	50%	100%
Sum:			4	-	80%	-	100%	-
Not answered:			1	-	20%	-	-	-
Average:	4.5	Minimum:		4		Variance:	0.33	
Median:	4.5	Maximum:		5		Std. deviation:	0.58	

Total answered: 4

Question 23

The feedback in the case is elaborated by explaining why something is right.

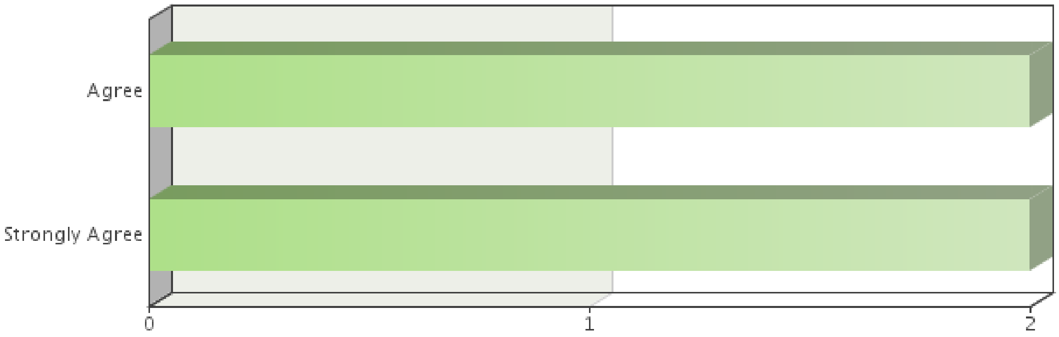

Frequency table

Levels			Absolute frequency	Cum. absolute frequency	Relative frequency	Cum. relative frequency	Adjusted relative frequency	Cum. adjusted relative frequency
Agree			2	2	40%	40%	50%	50%
Strongly Agree			2	4	40%	80%	50%	100%
Sum:			4	-	80%	-	100%	-
Not answered:			1	-	20%	-	-	-
Average:	4.5	Minimum:		4		Variance:	0.33	
Median:	4.5	Maximum:		5		Std. deviation:	0.58	

Total answered: 4

Question 24

The feedback in the case is elaborated by explaining why something is wrong.

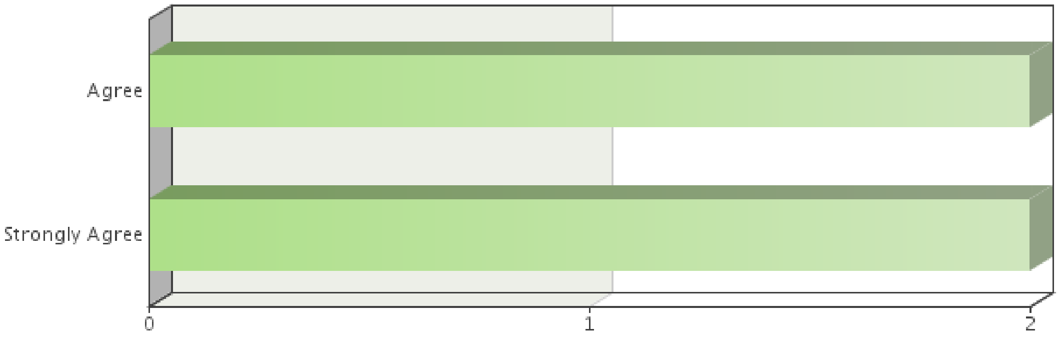

Frequency table

Levels	Absolute frequency	Cum. absolute frequency	Relative frequency	Cum. relative frequency	Adjusted relative frequency	Cum. adjusted relative frequency
Agree	2	2	40%	40%	50%	50%
Strongly Agree	2	4	40%	80%	50%	100%
Sum:	4	-	80%	-	100%	-
Not answered:	1	-	20%	-	-	-

Average:	4.5	Minimum:	4	Variance:	0.33
Median:	4.5	Maximum:	5	Std. deviation:	0.58

Total answered: 4

Question 25

The case offers possibilities for self-assessment.

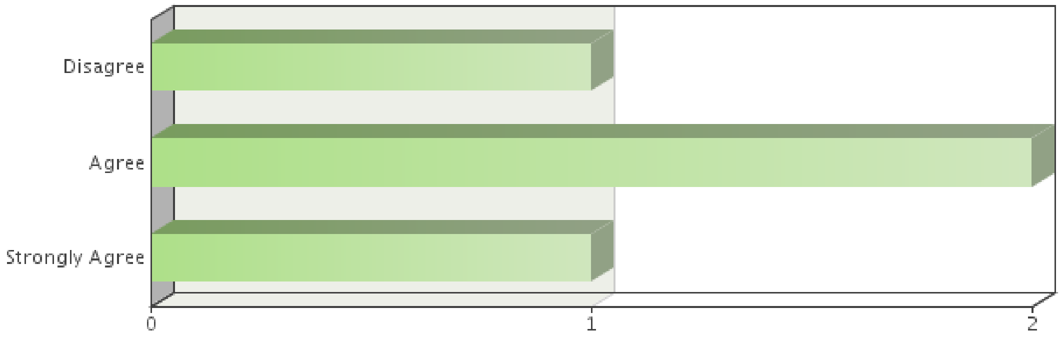

Frequency table

Levels	Absolute frequency	Cum. absolute frequency	Relative frequency	Cum. relative frequency	Adjusted relative frequency	Cum. adjusted relative frequency
Disagree	1	1	20%	20%	25%	25%
Agree	2	3	40%	60%	50%	75%
Strongly Agree	1	4	20%	80%	25%	100%
Sum:	4	-	80%	-	100%	-
Not answered:	1	-	20%	-	-	-

Average:	3.75	Minimum:	2	Variance:	1.58
Median:	4	Maximum:	5	Std. deviation:	1.26

Total answered: 4

Question 26

The case triggers the user (by e.g., prompts, feedback) to evaluate their actions of their inquiry.

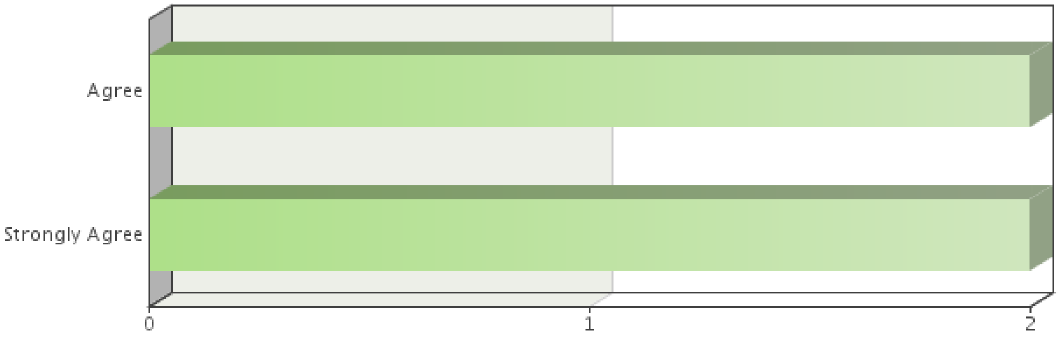

Frequency table

Levels			Absolute frequency	Cum. absolute frequency	Relative frequency	Cum. relative frequency	Adjusted relative frequency	Cum. adjusted relative frequency
Agree			2	2	40%	40%	50%	50%
Strongly Agree			2	4	40%	80%	50%	100%
Sum:			4	-	80%	-	100%	-
Not answered:			1	-	20%	-	-	-
Average:	4.5	Minimum:		4	Variance:		0.33	
Median:	4.5	Maximum:		5	Std. deviation:		0.58	

Total answered: 4

Question 27

The case triggers the user (by. e.g., prompts, feedback) to evaluate their diagnostic reasoning.

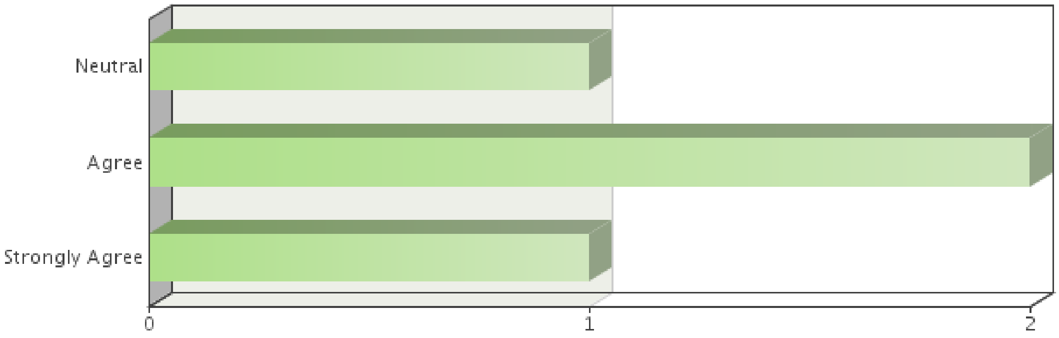

Frequency table

Levels	Absolute frequency	Cum. absolute frequency	Relative frequency	Cum. relative frequency	Adjusted relative frequency	Cum. adjusted relative frequency
Neutral	1	1	20%	20%	25%	25%
Agree	2	3	40%	60%	50%	75%
Strongly Agree	1	4	20%	80%	25%	100%
Sum:	4	-	80%	-	100%	-
Not answered:	1	-	20%	-	-	-

Average:	4	Minimum:	3	Variance:	0.67
Median:	4	Maximum:	5	Std. deviation:	0.82

Total answered: 4

Question 28

The case triggers the user (by. e.g., prompts, feedback) to evaluate their diagnostic reasoning.

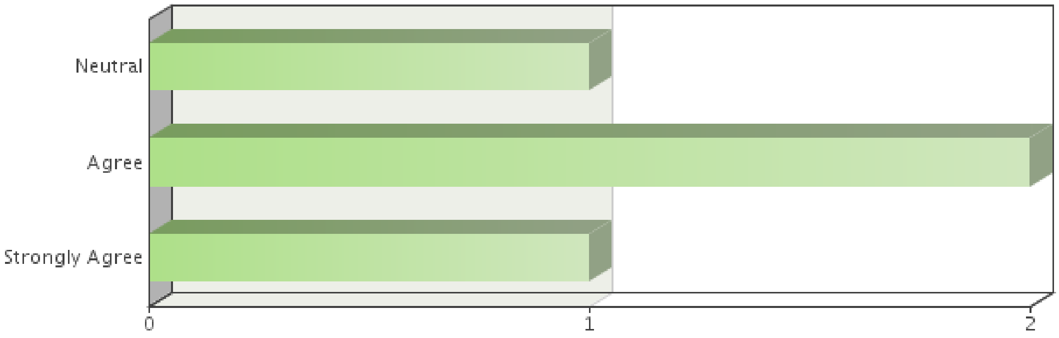

Frequency table

Levels		Absolute frequency	Cum. absolute frequency	Relative frequency	Cum. relative frequency	Adjusted relative frequency	Cum. adjusted relative frequency
Neutral		1	1	20%	20%	25%	25%
Agree		2	3	40%	60%	50%	75%
Strongly Agree		1	4	20%	80%	25%	100%
Sum:		4	-	80%	-	100%	-
Not answered:		1	-	20%	-	-	-
Average:	4	Minimum:	3	Variance:	0.67		
Median:	4	Maximum:	5	Std. deviation:	0.82		

Total answered: 4

Question 29

Overall, this case is very well suited to enhance learning in the target group.

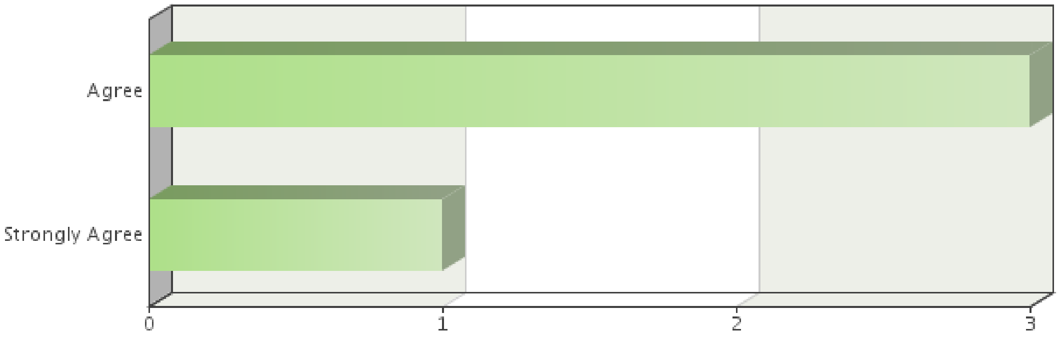

Frequency table

Levels	Absolute frequency	Cum. absolute frequency	Relative frequency	Cum. relative frequency	Adjusted relative frequency	Cum. adjusted relative frequency
Agree	3	3	60%	60%	75%	75%
Strongly Agree	1	4	20%	80%	25%	100%
Sum:	4	-	80%	-	100%	-
Not answered:	1	-	20%	-	-	-

Average:	4.25	Minimum:	4	Variance:	0.25
Median:	4	Maximum:	5	Std. deviation:	0.5

Total answered: 4

Question 30

Overall, this case is very well suited to foster clinical reasoning in the target group.

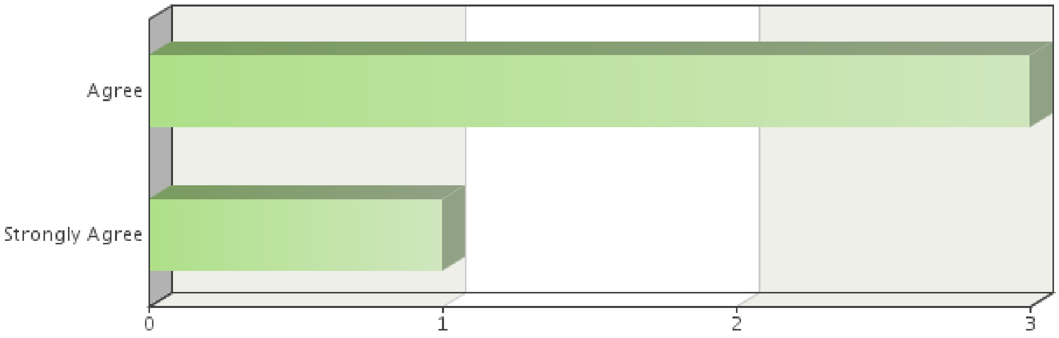

Frequency table

Levels			Absolute frequency	Cum. absolute frequency	Relative frequency	Cum. relative frequency	Adjusted relative frequency	Cum. adjusted relative frequency
Agree			3	3	60%	60%	75%	75%
Strongly Agree			1	4	20%	80%	25%	100%
Sum:			4	-	80%	-	100%	-
Not answered:			1	-	20%	-	-	-
Average:	4.25	Minimum:	4		Variance:	0.25		
Median:	4	Maximum:	5		Std. deviation:	0.5		

Total answered: 4

Question 31

Special weaknesses of this case

Question 32

Special strengths of this case
