# Supplementary material for: On-line virtual patient learning: a pilot study of a new modality in antimicrobial stewardship education for pediatric residents
Source: BMC Res Notes. 2020 Jul 14;13:339. doi: 10.1186/s13104-020-05170-7 (PMC7362648; doi:10.1186/s13104-020-05170-7)
Supplement: Supplementary file 3 — Additional file 3. Descriptive statistics of residents’ knowledge score Pre- and Post VP module. [file 13104_2020_5170_MOESM3_ESM.pdf]

**Supplementary file 3**

Table-1: Descriptive statistics of residents' knowledge score Pre- and Post VP module

| | Time | Mean (% correct answers) (SD) of Antibiotic Stewardship Knowledge score |
|---|---|---|
| **Junior Physicians** | **Time 1 = Pre-test (n=5)** | 52.00 (16.70) |
| | **Time 2= 1st Post-test (n=4)** | 65.5 (15.93) |
| | **Time 3= 4 months post-test (n=4)** | 69.75 (6.13) |
| **Senior Physicians** | **Time 1 = Pre-test (n=4)** | 63.20 (9.93) |
| | **Time 2= 1st Post-test (n=3)** | 67.60 (19.96) |
| | **Time 3= 4 months post-test (n=3)** | 73.20 (11.58) |
